# Pain Catastrophizing Beliefs and Neuropathic Symptoms Are Associated With a Poorer Long-Term Recovery in Chronic Plantar Heel Pain: A Cohort Study

**DOI:** 10.1093/ptj/pzaf134

**Published:** 2025-11-07

**Authors:** Jason Rogers, Graeme Jones, Karen Wills, Tania Winzenberg

**Affiliations:** Menzies Institute for Medical Research, University of Tasmania, Hobart, Tasmania, 7000, Australia; Menzies Institute for Medical Research, University of Tasmania, Hobart, Tasmania, 7000, Australia; Menzies Institute for Medical Research, University of Tasmania, Hobart, Tasmania, 7000, Australia; Menzies Institute for Medical Research, University of Tasmania, Hobart, Tasmania, 7000, Australia

**Keywords:** Cohort Studies, Fasciitis, Foot, Pain, Plantar, Podiatry, Prognosis, Risk Factors

## Abstract

**Importance:**

Chronic plantar heel pain is common and often recalcitrant yet understanding of modifiable risk factors that influence its trajectory of recovery is limited.

**Objective:**

The objective of this study was to describe associations of changes in physical and psychological measures and symptom descriptors over 12 months with changes in pain, function, and quality of life in people with chronic plantar heel pain.

**Design:**

A prospective cohort with longitudinal follow-up was used.

**Setting:**

A community setting in southern Tasmania was used.

**Participants:**

The participants were 220 people with a clinical diagnosis of chronic plantar heel pain.

**Exposures:**

The exposures were body mass index (kg/m^2^), waist circumference (centimeters), ankle plantarflexor strength (kilograms), ankle and first metatarsophalangeal joint dorsiflexion mobility (degrees), pain catastrophizing beliefs (Pain Catastrophizing Scale), depression (9-item Patient Health Questionnaire), multisite pain, morning stiffness, neuropathic symptoms (painDETECT), and physical activity (accelerometry).

**Main Outcomes and Measures:**

The Foot Health Status Questionnaire pain and function domains and the 6-dimension Assessment of Quality of Life Scale were used. Outcomes and exposures were assessed at baseline and 12 months. Data were analyzed using linear mixed–effects models with exposure × time interactions.

**Results:**

Increasing pain catastrophizing and neuropathic painDETECT scores over 12 months were associated with a poorer trajectory of pain recovery (pain catastrophizing interaction β = −.39 [95% CI = −0.01 to −0.77]; painDETECT interaction β = −.79 [95% CI = −0.10 to −1.48]). In full multivariable models, there were no other significant associations between any other variable and pain. The only associations with foot function and quality of life were weak negative associations of steps per day and sedentary time with function and quality of life, respectively.

**Conclusions and Relevance:**

Increasing pain catastrophizing and neuropathic symptoms were associated with poorer pain outcomes over 12 months in individuals with chronic plantar heel pain. These findings highlight the importance of pain beliefs and neurogenic factors in the prognosis of chronic plantar heel pain. Interventions targeting pain beliefs and neuropathic mechanisms may improve outcomes in subgroups with these characteristics.

## INTRODUCTION

Chronic plantar heel pain (CPHP) is commonly assumed to have a self–limiting natural course.^1^ This view is based predominantly on retrospective case series analyses^2^^,^^3^ or control–arm trial data, which is not explicitly designed to report on longitudinal outcomes in CPHP.^4–6^ In contrast, in a recent prospective study as many as 46% of participants reported mild but ongoing symptoms an average of 10 years after onset.^7^ This suggests that the persistence of CPHP may be underestimated and pathways to recovery may not be the same for all. Therefore, understanding what factors are associated with the course of the disease is important.

Improving our understanding of factors affecting CPHP outcomes could help identify potential causal mechanisms and allow treatments to be directed towards factors likely to matter. Currently identified prognostic factors for CPHP are restricted to a few clinical, demographic or disease factors such as bilateral symptoms,^3^^,^^7^ female sex,^7^ overweight,^3^ and longer duration of symptoms.^3^ Most are not modifiable and are therefore of limited clinical value. Other clinical factors with an established cross-sectional association,^8^ such as pain catastrophizing, depression, calf strength, or multisite pain have not been evaluated for longitudinal associations. Even less is known about how different pain mechanisms impact CPHP. Direct damage to the nociceptive system (neuropathic mechanism) or altered nociception due to adaptations within nociceptive pathways of the nervous system (nociplastic mechanism) may operate predominantly or in conjunction with locally generated nociceptor activation.^9^ For example, symptoms associated with nerve injury in CPHP may affect up to 1 in 5 cases.^10^ This is an important consideration for clinicians as neuropathic symptoms are associated with poorer clinical outcomes in a range of other musculoskeletal pain states such as low back pain and osteoarthritic knee pain.^11^ Many of these under-investigated factors are potentially modifiable; improving understanding of them could help guide clinical decision-making.

The primary aim of this prospective cohort study therefore was to determine how change in clinical factors, assessed by psychological survey, physical measures, and symptom descriptors, are associated with change in CPHP pain, function and quality-of-life outcomes over 12 months.

## METHODS

### Setting and Participants

Participants with CPHP were recruited in southern Tasmania between November 2014 and May 2016. To maximize representation we recruited from medical and allied health practices, newspaper advertising, social media, sporting clubs, community organizations, and hospital and government workplaces.^8^ Participants were invited back for follow-up 1 year later, with reassessments taking place between December 2015 and August 2017.

The study was approved by the University of Tasmania Health & Medical Human Research Ethics Committee (H0013616). All participants provided written informed consent.

### Inclusion/Exclusion

Participants were aged 18 years and older with a clinical diagnosis of CPHP defined by pain under the heel that is aggravated by weight-bearing function^8^ and has lasted for at least 3 months. If heel pain was bilateral, the most symptomatic heel was assessed. Potential participants at baseline were excluded if they had a history of previous foot/ankle fracture or orthopedic foot surgery, current ankle pain, recent foot trauma, or other orthopedic, congenital, vascular, neurological, or painful lower limb condition that restricted mobility or activity in the preceding 3 months. Participants who had a corticosteroid or any other injection, shock wave treatment, or steroid iontophoresis within the previous 6 months were excluded. As we had an imaging arm attached to this study, any participant with a contraindication to MRI was also excluded.

### Sample Size Calculations

Sample size was calculated assuming α = .05 (2-tailed) and 80% power for longitudinal hypotheses, with a loss to follow-up of 10%. Assessing the association between exposures and the primary outcome of Foot Health Status Questionnaire (FHSQ) pain, a sample size at baseline of 220 allows for the detection of at least small correlations (≥0.2; variance = 4%).

### Data Collection for Outcomes

Outcomes were collected at baseline and 12 months. Foot pain and foot function were assessed by the pain and function domains of the FHSQ (Cronbach α = .86; ICC = 0.92).^12^ The FHSQ provides a region specific measure of foot health status on a weighted continuous scale ranging from 0 to 100, where higher scores represent less pain and better function.^12^ Quality of life was measured with the 6-dimension Assessment of Quality of Life Scale (Cronbach α = .94; ICC = 0.85–0.88).^13^ The 6-dimension Assessment of Quality of Life Scale is a 20-item multi-attribute instrument used to calculate an overall quality-of-life score ranging from 0 to 100, where higher is better, on the basis of the sum of all unweighted responses.

### Data Collection for Exposures

Exposures were measured at baseline and 12 months as previously published.^8^ Clinical measures were taken in a single session at both time points by the same experienced physical therapist. Accelerometry and survey measures were collected and entered by research assistants. The physical therapist who collected clinical data conducted the analyses on data de-identified by ID number.

Our key exposures of interest based on factors considered important from previous research were body mass index (BMI)/waist girth, pain catastrophizing beliefs, multisite pain, and ankle plantarflexor strength, to which we also added the painDETECT neuropathic pain score.^8^ To calculate the BMI (weight [kg]/height [m]^2^), height was measured to the nearest 0.1 cm using a stadiometer, and weight was measured to the nearest 0·1 kg with a single set of calibrated scales (UC321-PL; A&D Medical, Adelaide, South Australia, Australia). Waist girth was measured to the nearest millimeter in the horizontal plane with steel tape (model W606PM; Crescent Lufkin, Sparks, MD, USA) at the midaxillary midpoint between the iliac crest and the twelfth rib; the study ICC(3,1) was 0.98.^14^ Maximum isometric ankle plantarflexor strength was measured in the sitting position as the highest score from 3 attempts with the lower limb strapped by a nonelastic belt about the knee to a digital scale (Excell GW; Excell Precision Co, Ltd, New Taipei City, Taiwan); the study ICC(3,1) was 0.96.^15^ Pain beliefs were measured using the Pain Catastrophizing Scale,^16^ with a score of >20 being considered as clinically important pain catastrophizing.^17^ Multisite pain was recorded by checklist as the sum of body region pain sites other than the heel ranging from 0 (no other sites) to 7, considering bilateral symptoms as a single contribution**.** The presence of neuropathic symptoms was assessed using the painDETECT questionnaire.^11^ This questionnaire has a score range of −1 to 38, with scores of 19 or more indicating a neuropathic component to pain.^11^

Other clinical, physical activity and symptom or survey measures were considered as covariates or secondary exposures of interest. Ankle dorsiflexion (degrees) was measured with a gravity inclinometer (Plurimeter; Dr Rippstein, Zurich, Switzerland) placed on the mid-anterior shin in a weight–bearing lunge position with the knee flexed (ICC ≥ 0.88).^18^ Passive first metatarsophalangeal joint extension (degrees) was measured with the foot plantigrade in the supine position as the mean of 3 goniometric measurements; the study ICC(3,1) was 0.95.^19^ Physical activity was measured with a uniaxial accelerometer (ActiGraph GT1M; ActiGraph, Fort Walton Beach, FL, USA) worn at the waist for 7 consecutive days. Steps per day and mean counts per minute were measured, and physical activity classified as minutes spent in moderate to vigorous, light, and sedentary activities. Criteria for acceptable wear time, activity thresholds and data processing have been published previously.^8^

Questionnaires recorded age, sex, smoking history, presence of morning stiffness symptoms (at any joint), and comorbidities (diabetes or rheumatological disease). Symptoms of depression were measured with the 9-item Patient Health Questionnaire (PHQ-9).^20^ Pain laterality, duration of symptoms, and number of previous episodes were also recorded. Self-reported treatments received during the follow-up period were assessed by questionnaire. Participants could check “yes” to any of 27 explicitly listed treatment options or provide a free-text response for any other treatment received but not listed.

### Data Analysis

Linear mixed–effects models were used to estimate whether change in each clinical exposure over 12 months affected change in pain, function, and quality-of-life outcomes over 12 months. To account for correlated observations, all models included a random intercept for individuals, specifying an unstructured covariance structure**.** We did not include random slope models as it is not possible to estimate random slope variance when only 2 time points are available.^21^

We fit separate linear mixed–effects models for each time-varying outcome, including fixed effects for the time–varying clinical exposure, time (treated as a categorical variable: baseline/follow-up), an exposure × time interaction term, age, sex, and other model-specific adjustors. The interaction term was included to test whether the effect of the exposure on the outcome differed between baseline and follow-up, consistent with our aim to assess change over time.

Our primary exposures of interest in model building, developed from our earlier work, were BMI/waist girth, pain catastrophizing beliefs, multisite pain, and ankle plantarflexor strength.^8^ We also considered neuropathic symptoms based on the painDETECT score as an exposure of interest, which could not be analyzed in our prior case–control analysis given the symptom-free status of controls.

In the first stage of model building, we fitted simple age- and sex-adjusted models separately for each exposure along with an exposure × time interaction term. In subsequent models we further adjusted for current smoking status, physical activity (accelerometry) and the presence of comorbidities (diabetes and inflammatory disease separately). We did not adjust for diabetes in our BMI model as they are potentially on the same causal pathway. Similarly, we did not adjust for physical activity in our pain catastrophizing or neuropathic pain models model as a behavioral consequence of pain and pain catastrophizing can be fear of movement and reduced physical activity. For physical activity, we considered that a measure of sedentary time was more appropriate for BMI, waist girth, and multisite pain models but that vigorous activity was more appropriate in a model analyzing ankle strength, which was also adjusted for body weight. We analyzed multisite pain in continuous form (0–7) to minimize the risk of sparse data bias. As a sensitivity analysis we adjusted for treatments received that reflect recommended practice guidelines.^22^ This included having received any 1 or more of stretching the plantar fascia, taping, orthoses, injections, or shock wave treatment in the period between baseline and follow-up assessments.

Models were checked for multicollinearity and information criteria (Akaike information criterion/Bayesian information criterion) were used to assess model fit. As linear mixed–effects models account for missing outcome data but not missing exposure data, we used inverse probability weighting to account for the latter, assuming data were missing at random with observations weighted by the inverse of their probability of being a complete case. This probability was estimated via logistic regression using baseline demographic and clinical predictors. Test significance was set at *P* < .05.

Model assumptions were checked by examining residuals for normality and constant variance. Linearity was checked using fractional polynomials. If meaningful clinical categorizations were available, we also expressed output in that form if model fit by information criteria indicated at least as good a fit as the linear term. All analyses were performed using Stata 18 (Stata Corp, College Station, TX, USA).

### Patient and Public Involvement

Patients or members of the public were not involved in the planning, design, or implementation of this study. Patients were not invited to contribute to interpretation of the results or the writing of this document.

### Role of the Funding Source

The funders played no role in the design, conduct, or reporting of this study.

## RESULTS

**Figure 1:**
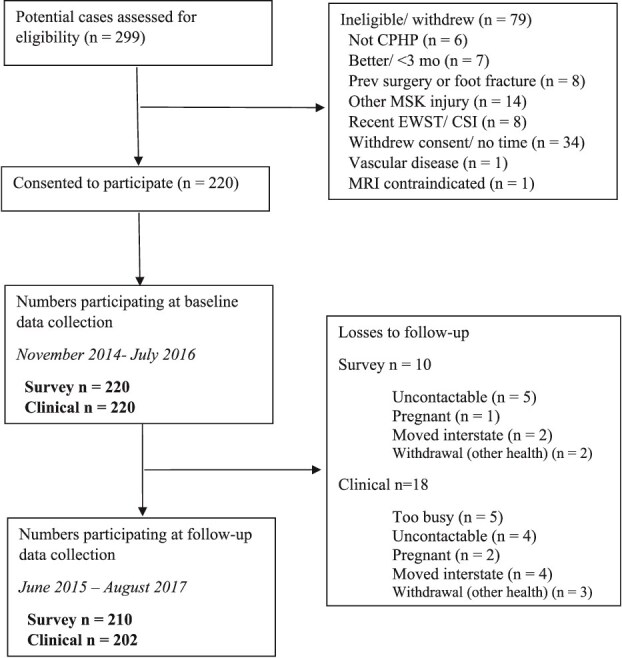
Participant Flow. Abbreviations: CPHP = chronic plantar heel pain; CSI = corticosteroid injection; EWST = extra corporeal shockwave therapy; MRI = magnetic resonance imaging; MSK = musculoskeletal; Prev = previous.

Of 220 participants assessed at baseline, 210 returned surveys and 202 attended clinical reassessment a minimum of 1 year later (Figure 1). The median time to follow-up was 406 days (interquartile range = 373-430 days).

From baseline to follow-up, mean FHSQ pain scores increased from 48.8/100 to 75.9/100, indicating a decrease in pain. Foot-related function improved by 31% (from 65.7–86.13), and AQoL-6D scores improved 7% (from 76.4–81.6) (Table 1). Twenty-one percent of participants reported no pain at follow-up (FHSQ = 100/100), and 67% reported pain that improved by at least the minimal important difference (MID) (pain domain, 13 points).^23^ Twenty-one percent of participants self-reported currently receiving treatment at baseline, compared to 7% at follow-up. Table 2 lists the types and frequency of treatments participants reported receiving in the period between baseline and follow-up assessments. The most common treatments were stretching the calf/muscles of the lower leg, stretching the plantar fascia, and activity modification (resting or stopping activity). Participants who did not return outcome measures at follow-up had worse baseline pain catastrophizing and painDETECT scores, although the loss to follow-up for those measures was small (5%) (Suppl. Table 1).

**Table 1:** Characteristics of Participants at Baseline and Follow-up*^a^*

**Characteristic**	**Baseline**		**Follow-up**	
	**Value**	**Total no. of participants**	**Value**	**Total no. of participants**
FHSQ pain score/100, mean (SD)	48.8 (21.6)	220	75.9 (23.3)	210
Clinically important pain improvement*^b^*			67 (140)	210
FHSQ function score/100, mean (SD)	65.7 (27.8)	220	86.13 (22.3)	210
AQoL-6D score/100, mean (SD)	76.4 (10.8)	220	81.6 (9.7)	209
Women	60 (131)	220	60 (126)	210
Age, y, mean (SD)	54.8 (12.13)	220	56.0 (12.04)	211
Ever having smoked	33 (73)	220	31 (66)	210
Diabetes	4 (8)	218	4 (8)	202
Inflammatory disease	9 (18)	207	10 (20)	201
High cholesterol	25 (55)	220	23 (49)	209
Osteoporosis/osteopenia	12 (7)	58	13 (8)	63
Morning stiffness	22 (48)		14 (29)	
Multisite pain	81 (179)	220	81 (170)	210
BMI, kg/m^2^, mean (SD)	29.1 (5.4)	220	29.5 (5.5)	202
Ankle DF ROM with knee flexed, °, mean (SD)	43.3 (6.7)	220	43.3 (7.0)	202
Ankle PF strength, kg, mean (SD)	90.5 (23.8)	220	91.2 (24.6)	201
First MTPJ extension ROM, °, mean (SD)	70.3 (15.1)	220	71.7 (16.4)	202
PHQ-9 depression score/27, median (IQR)	2 (1–5)	220	2 (0–5)	209
Moderately depressed, ≥10*^c^*	16 (34)	220	11 (22)	209
painDETECT score/38, median (IQR)	9 (5–13)	220	3 (0–8)	209
“Probably neuropathic,” ≥19*^d^*	8 (17)	220	5 (10)	210
PCS catastrophizing score/52, median (IQR)	8 (4–16)	220	3 (0–9)	209
Catastrophizing, >20*^e^*	17 (38)	220	7 (15)	209
Physical activity, median (IQR)				
Average steps/d	7760 (6046–9871)	211	7702 (5837–9839)	182
MVPA, min/d	37.7 (16.9–55.4)	211	32.0 (18.3–61.1)	198
Sedentary time, min/d	491 (437–545)	211	507 (442–551)	183

^a^ Data are reported as percentages (numbers) of participants unless otherwise indicated. Abbreviations: AQoL-6D = 6-dimension Assessment of Quality of Life Scale (scored from 0–100; higher = better); BMI = body mass index; DF = dorsiflexion; FHSQ = Foot Health Status Questionnaire (scored from 0–100; higher = better); IQR = interquartile range; MTPJ = metatarsophalangeal joint; MVPA = moderate to vigorous physical activity; PCS = Pain Catastrophizing Scale (higher = worse); PF = plantarflexion; PHQ-9 = 9-item Patient Health Questionnaire (higher = worse); ROM = range of motion.

^b^ FHSQ pain improvement at or exceeding the minimal important difference (≥13 points).^23^

^c^ Moderately depressed cutoff point (Kroenke et al, 2001).^24^

^d^ “Probably neuropathic” cutoff point.^11^

^e^ Clinical catastrophizing cutoff point.^25^

**Table 2:** Treatments Received in the Year between Baseline and Follow-up Assessments

**Treatment Received During the Year** ^***a***^	**No. (%) of Participants**
Resting/stopping activity	95 (43)
Stretching lower leg	125 (57)
Stretching arch	114 (52)
Massaging lower leg	82 (37)
Massaging arch	93 (42)
Strengthening leg	60 (27)
Strengthening foot	54 (25)
Other exercise	32 (15)
Manual therapy	30 (14)
Acupuncture	25 (11)
Orthotics, custom	68 (31)
Orthotics, over-the-counter	69 (31)
Heel cups	46 (21)
Footwear	86 (39)
Taping	53 (24)
Night splints	15 (7)
Steroid injection	9 (4)
Blood injection	2 (1)
Iontophoresis	5 (2)
Shock wave therapy	4 (2)
Surgery	1 (0)
Prescription medication	23 (10)
Over-the-counter medications	33 (15)

^a^ Participants responded “yes” (Y) or “no” (N) to the statement, “Select as many options as appropriate to reflect any and all treatment choices you have tried in the past year.” Checklist and free-text options were available.

Results for simple age- and sex-adjusted and full multivariable adjusted models for primary exposures are presented in Table 3. Effects are reported in the table of results as beta coefficients for a (conditional) main effect for the exposure of interest, an effect for time, and an exposure × time interaction term. For continuous measures, the main effect identifies the change in outcome per unit of exposure at baseline. The coefficient for time indicates the average change in outcome over time taken at the baseline or reference level of exposure, and the exposure × time interaction term indicates the change in outcome over time for a 1-unit increase in the exposure. For context, effects are considered relative to the MID for that outcome (where known) in the scale of the outcome. Effects that exceeded the MID were considered large, those approaching the MID were considered medium, and those near 0 were considered small to none.

**Table 3:** Simple and Full Multivariable Models for Outcomes of Pain, Function, and Quality of Life*^a^*

**Parameter**	**FHSQ Pain** ^***b***^						**FHSQ Function** ^***c***^						**AQoL-6D** ^***d***^					
	**Age and sex** ^***e***^			**Full multivariable** ^***f***^			**Age and sex**			**Full multivariable**			**Age and sex**			**Full multivariable**		
	**β**	**95% CI**		**β**	**95% CI**		**β**	**95% CI**		**β**	**95% CI**		**β**	**95% CI**		**β**	**95% CI**	
		**Lower Limit**	**Upper Limit**		**Lower Limit**	**Upper Limit**		**Lower Limit**	**Upper Limit**		**Lower Limit**	**Upper Limit**		**Lower Limit**	**Upper Limit**		**Lower Limit**	**Upper Limit**
BMI (kg/m^2^)	**−.98**	−1.47	−0.48	**−1.02**	−1.51	−0.52	**−1.54**	−2.20	−0.88	**−1.50**	−2.15	−0.85	**−.71**	−0.96	−0.45	**−.67**	−0.92	−0.42
Time	**27.18**	23.55	30.80	**26.74**	23.10	30.39	**20.63**	17.34	23.92	**19.40**	15.95	22.85	**5.18**	4.24	6.12	**5.15**	4.18	6.12
BMI × time*^g^*	*.57*	−0.08	1.22	.49	−0.20	1.17	**.77**	0.11	1.43	.55	−0.17	1.28	.17	−0.01	0.35	.16	−0.02	0.35
Waist (cm)	**−.38**	−0.59	−0.17	**−.38**	−0.60	−0.18	**−.58**	−0.86	−0.29	**−.58**	−0.86	−0.31	**−0.26**	−0.36	−0.15	**−.25**	−0.35	−0.14
Time	**26.58**	22.94	30.22	**25.97**	22.32	29.63	**19.67**	16.38	22.97	**18.24**	14.78	21.70	**4.68**	3.71	5.65	**4.61**	3.62	5.60
Waist × time*^g^*	.18	−0.07	0.43	.14	−0.12	0.39	*.26*	−0.01	0.52	.19	−0.11	0.48	.04	−0.03	0.12	.04	−0.04	0.11
painDETECT*^h^*	**−1.45**	−1.89	−1.01	**−1.43**	−1.93	−0.94	**−1.77**	−2.27	−1.27	**−1.51**	−2.04	−0.97	**−.47**	−0.63	−0.30	**−.51**	−0.69	−0.34
Time	**23.96**	18.83	29.09	**23.81**	18.31	29.31	**11.03**	5.92	16.14	**12.39**	7.02	17.76	**2.19**	0.60	3.77	**1.86**	0.22	3.50
painDETECT × time*^g^*	**−.73**	−1.32	−0.13	**−.79**	−1.48	−0.10	.22	−0.34	0.78	−.06	−0.74	0.62	.11	−0.07	0.30	*.18*	0.00	0.36
PCS*^i^*	**−.85**	−1.13	−0.56	**−.80**	−1.14	−0.47	**−1.31**	−1.63	−0.98	**−1.42**	−1.74	−1.10	**−.47**	−0.57	−0.37	**−.47**	−0.58	−0.36
Time	**24.09**	19.84	28.35	**24.77**	20.45	29.09	**13.18**	9.07	17.29	**12.45**	9.07	17.29	**2.65**	1.46	3.83	**2.81**	1.58	4.04
PCS × time*^g^*	−.22	−0.63	0.19	**−.39**	−0.77	−0.01	.08	−0.35	0.51	.01	−0.41	0.42	−.01	−0.14	0.11	.00	−0.13	0.13
Multisite pain*^j^*	−.98	−2.37	0.42	−.62	−2.11	0.86	**−2.46**	−4.08	−0.83	**−2.44**	−4.26	−0.61	**−.87**	−1.45	−0.30	**−1.16**	−1.79	−0.52
Time	**30.79**	24.93	36.64	**27.95**	22.00	33.91	**19.40**	14.19	24.62	**16.62**	11.26	21.99	**4.88**	3.36	6.41	**4.19**	2.62	5.76
Multisite × time*^g^*	−1.54	−3.41	0.33	−.63	−2.55	1.30	.37	−1.34	2.08	1.10	−0.94	3.15	.02	−0.49	0.53	.35	−0.22	0.92
Plantarflexor strength (kg)	**.16**	0.01	0.30	**.19**	0.05	0.34	**.32**	0.13	0.51	**.33**	0.15	0.51	.04	−0.02	0.11	**.07**	0.01	0.13
Time	**32.99**	17.89	48.09	**30.21**	15.70	44.72	**33.82**	17.69	49.94	**28.36**	12.85	43.88	2.67	−2.36	7.71	3.27	−2.36	7.71
Strength × time*^g^*	−.07	−0.23	0.10	−.04	−0.19	0.11	*−.15*	−0.33	0.02	−.10	−0.27	0.06	.02	−0.03	0.08	.02	−0.03	0.07

^a^ Abbreviations: AQoL-6D = 6-dimension Assessment of Quality-of-Life Scale; β = β coefficient; BMI = body mass index; FHSQ = Foot Health Status Questionnaire; PCS = Pain Catastrophizing Scale.

^b^ Scored from 0 to 100; higher scores indicate less pain.

^c^ Scored from 0 to 100; higher scores indicate better function.

^d^ Scored from 0 to 100; higher scores indicate better quality of life.

^e^ Simple multivariable models were adjusted for age and sex. Inverse probability weighting applied for missing data; bold type indicates significance at *P* < .05; italic type indicates *P* < .10.

^f^ Full multivariable models were adjusted as follows: BMI and waist girth models were adjusted for age, sex, inflammatory disease comorbidities, sedentary time (min/d), and current smoking; painDETECT and pain catastrophizing models were adjusted for age, sex, comorbidities, and current smoking; the multisite pain model was adjusted for age, sex, sedentary time (min/d), comorbidities, and current smoking; and the ankle plantarflexor strength model was adjusted for age, sex, moderate to vigorous physical activity (min/d), and body weight. Inverse probability weighting applied for missing data; bold type indicates significance at *P* < .05; italic type indicates *P* < .10.

^g^ Exposure × time interaction term.

^h^ painDETECT was scored from −1 to 38; higher scores more likely represent neuropathic pain.

^i^ PCS was scored from 0 to 52; higher scores represent greater catastrophizing.

^j^ No. of sites of bodily pain by region other than the heel, modelled as continuous (scored from 0–7).

**Figure 2:**
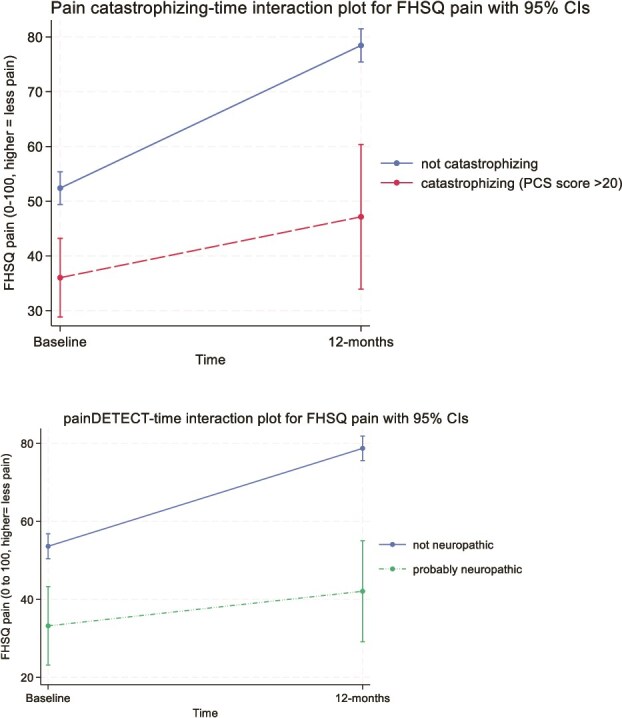
Full multivariable model interaction plots for pain catastrophizing (top) and painDETECT (neuropathic pain) (bottom) for the outcome of pain. Pain catastrophizing scores are categorized as “catastrophizing” on the basis of a Pain Catastrophizing Scale (PCS) score of >20. painDETECT scores are classified as “probably neuropathic” on the basis of a painDETECT score of ≥19. Categorization is based on exposure status at each time point. The x-axis is time (baseline or follow-up). The y-axis is FHSQ pain scored from 0 to 100, where higher scores reflect less pain. In both instances, higher PCS and painDETECT scores were associated with worse baseline pain and a poorer trajectory of pain recovery over 12 months. ^*^Continuously plotted interactions for pain catastrophizing and painDETECT for outcome of pain also presented in Supplementary Figure 1.

In simple age- and sex-adjusted models, an increase in painDETECT score was associated with poorer improvement in pain over 12 months (interaction β = −.73 [95% CI = −0.13 to −1.32]). In fully adjusted multivariable models, an increase in both painDETECT and pain catastrophizing scores were associated with less improvement in pain over 12 months (interaction β = −.79 [95% CI = −0.10 to −1.48] and interaction β = −.39 [95% CI = −0.01 to −0.77], respectively). For both measures, higher scores were associated with worse baseline pain that improved less over 12 months when categorized as “catastrophizing” (Pain Catastrophizing Scale score > 20) or as “probably neuropathic” on painDETECT (≥19) (Figure 2). People classified as catastrophizing had less pain improvement than those who were not (interaction β = −15 [95% CI = −28.5 to −1.4]); the same was true for those classified as “probably neuropathic” rather than “not neuropathic” (interaction β = −15.8 [95% CI = −29.9 to −1.7]). Both effects were considered large enough to be clinically relevant, as they exceeded the known MID for FHSQ pain, albeit with wide CIs. There were no other significant interactions in full multivariable models for pain.

In simple age- and sex-adjusted models for foot function, there was a small interaction effect for BMI; a 1-unit increase in BMI over time was associated with a 0.77-unit improvement in FHSQ function (95% CI = 0.11–1.43), but this finding did not persist in the fully adjusted model. In multivariable models, none of the primary exposures were significantly associated with foot function or quality of life over time. Interaction plots for each outcome for the painDETECT, pain catastrophizing, waist girth, BMI, ankle plantarflexor strength, and multisite pain are presented in Supplementary Figures 1-3.

Results for secondary exposures are given in Supplementary Table 2. Other than small negative associations of average steps per day (out of 1000 steps) with function (interaction β = −1.50 [95% CI = −2.89 to −0.11]) and of moderate to vigorous physical activity (min/d) with quality of life (interaction β = −.04 [95% CI = −0.07 to −0.01]), none of these exposures were associated with any outcome. Adjusting for treatments received did not change our conclusions for any outcome based on coefficient estimates or significance (data not shown).

## DISCUSSION

Pain outcomes over 12 months in people with CPHP, but not function or quality of life, were poorer in people with worsening catastrophizing beliefs or neuropathic symptoms. Other factors considered potentially important in CPHP risk or prognosis, including BMI, waist girth, ankle plantarflexor strength, and multisite pain, were not significantly associated with longitudinal CPHP outcomes in multivariable models. An association with pain beliefs and pain mechanisms indicates that person-level factors beyond the foot are important considerations in CPHP. High-quality interventional research is required to determine if targeting these potentially modifiable phenotypes can improve treatment outcomes for CPHP.

### Neuropathic Symptoms

The novel finding for CPHP that increasing neuropathic symptoms are associated with poorer pain outcomes over 12 months indicates that non-nociceptive pain mechanisms are important in a sub-group of individuals with CPHP. These results highlight the mechanistic role neuropathic pain may play in long–term CPHP pain outcomes, indicating promise for treatment options that can modify its trajectory. People with pain categorized as “probably neuropathic” on painDETECT (≥19) also had worse baseline pain suggesting this measure might assist clinicians to identify people for whom such treatments might be an option. For clinicians, identifying neuropathic heel pain is notoriously difficult, yet original case reports have found electrodiagnostic abnormalities in 15%-20% of cases^10^ with numerous sites for potential nerve injury identified.^26^ For the painDETECT we used the traditional cutoff point of >19 to identify 8% of participants as “probably neuropathic”. This measure seeks to differentiate nociceptive from neuropathic pain and was validated in a low back pain population, referenced to expert opinion.^11^ It was developed at a time before the concept of nociplastic pain, that is pain arising from altered nociception despite no clear evidence of actual or threatened tissue damage or lesion in the somatosensory system,^27^ had been formalized. This scale therefore likely captures features of both pain types, and could signpost the need for clinicians to consider further assessment of non-nociceptive features (eg, widespread symptoms, symptom gain, non-anatomic distribution). Other tools measuring symptoms of sensitization such as the central sensitization inventory,^28^^,^^29^ could also be considered. The few studies that have investigated painDETECT in a CPHP population used these previously established cutoff points. Although they demonstrate that neuropathic pain by this criteria is prevalent^30^ and associated with having CPHP,^8^ the diagnostic performance of this scale and its thresholds have not been tested in CPHP. It is also yet to be established whether phenotyping CPHP by pain subtypes is clinically useful. However, given that treatment approaches for these conditions are not routinely catered for in standard CPHP guidelines, this offers a promising area for further study.

### Pain Catastrophizing Beliefs

Pain catastrophizing beliefs are longitudinally associated with a poorer prognosis for pain outcomes in CPHP. When categorized as “clinical catastrophizing”^25^ the effect size exceeds the known MID for this pain scale^23^ indicating that it is likely clinically important (Figure 2, top). Pain catastrophizing describes an exaggerated negative mental state about pain or anticipated pain and reflects a cognitive appraisal dominated by a sense of helplessness, ruminating thoughts and symptom magnification.^16^ Although case-control and cross-sectional associations of pain catastrophizing with CPHP have been previously demonstrated,^8^^,^^31^ and a longitudinal association between catastrophizing and pain severity and chronicity established for other musculoskeletal conditions,^32^ this is the first study to confirm a longitudinal association in CPHP. Pain beliefs are a potentially modifiable driver of pain in CPHP. It can be quantified and catastrophizing status determined using the pain catastrophizing scale to help direct treatment choices, although the optimal threshold for action in CPHP is unknown. Effective treatment of pain in people with dominant negative pain beliefs requires addressing the underlying cognitive mechanisms. Treating pain alone may be insufficient as the effect of pain beliefs is independent of pain severity.^33^ Novel treatments for CPHP could therefore include cognitive strategies to reconceptualize pain, which has been shown to be effective at reducing pain catastrophizing.^34^

### Other Factors

Despite associations with pain, neither pain beliefs, neuropathic symptoms, or BMI (other than in the simple age and sex adjusted model for function) were significantly associated with function or quality-of-life outcomes. Function and quality-of-life measures changed less from baseline to follow-up, from a relatively better starting point. Potential ceiling effects, and reduced variability may make detecting change more difficult for these outcomes. As distinct from case-control findings,^8^ change in multisite pain, ankle plantarflexor strength, and waist girth were also not associated with any CPHP outcome over 12 months. This may be because case-control results reflect a non-causal association, with findings potentially subject to bias due to unmeasured confounding. Alternatively, these factors may be better at describing “risk” for onset rather than chronicity.

For other variables, taking more steps/day being associated with a poorer recovery of function, and increasing minutes per day of moderate to vigorous physical activity was associated with a poorer quality of life (Suppl. Table 2). Although contrary to expectation, inspecting the interaction plots (Suppl. Figure 4) notes that higher quantities of both these measures are associated with better function and quality of life at baseline. The negative interaction creates a “catch up” phenomenon that possibly reflects regression to the mean, with similar outcomes for all levels of physical activity at follow-up. For other measures such as symptoms of morning stiffness, depression and for common clinical measures of ankle, hallux, or straight leg raise mobility, no significant effect over time was found for any outcome (Suppl. Table 2). These results are consistent with our previous case-control findings and collectively, may challenge the value of addressing these impairments once disease is established.

### Strengths and Limitations

The strengths of this study are the large case sample, high follow-up rate, and detailed longitudinal exposure set including for the first-time in CPHP neuropathic and psychological factors, physical activity and many of our clinical measures. The large case sample, inclusive case definition and community-wide recruitment supports the generalizability of our results. Nonetheless, there are also limitations. Although difficult to avoid, some of our key exposures rely on subjective self-report, and given the observational nature of this study, may be subject to reporting bias. A prospective longitudinal cohort strengthens the case for understanding causal mechanisms but is not proof of causation. Examining change in exposure over an extended period with a single follow-up may miss variability in trajectories, especially for time-varying predictors. Although cost and resources precluded it, intermediate time points would have been helpful to further categorize differential rates of change, and to help understand the short-term effects of change in trajectory, which may have greater clinical utility. Intermediate time points would also allow for the assessment of incident pain in the test foot following resolution. It cannot be discounted that follow up reporting captures some participants with new pain, noting that defining when an episode starts and stops is not straightforward. The a priori sample size calculations were based on correlation size. This did not fully account for the complexity of linear mixed-effects modeling, including within-subject correlation and random-effects structure.^35^^,^^36^ This may have underestimated the required sample size, resulting in reduced power and loss of estimate precision. Future studies should employ sample size calculations specifically designed for mixed-effects models, such as those implemented in tools like G*Power or PASS.^37^ Lastly, we collected limited treatment data, by self-report only. This restricts our ability to differentiate the contribution of natural history versus treated course to outcomes, including the potentially important effect of treatment expectations.^38^ A more detailed assessment of treatments received, treatment expectations and in the case of longitudinal observational studies, expectations around disease trajectory, could inform future studies.

### CONCLUSIONS

Pain catastrophizing beliefs and neuropathic symptoms that increase over time distinguish subgroups of people with CPHP who have a poorer trajectory of pain recovery. These findings highlight the important role of psychological factors and pain mechanisms in CPHP including the need for clinicians to identify factors beyond the foot when developing individualized treatment plans. Future research should determine if targeting pain beliefs and neuropathic symptoms in sub-groups identified with these impairments, respond to pain science and neuropathically informed interventions, above and beyond standard care.

## Supplementary Material

PTJ-2025-0165_R2-Supplementary-Material_JR_pzaf134

## Data Availability

The data generated from this study will not be deposited in a public repository due to privacy and consent restrictions. De-identified data in Excel spreadsheet form, including survey and clinical scores, are available from the corresponding author on reasonable request, subject to a data sharing agreement.

## References

[1] Landorf KB . Plantar heel pain and plantar fasciitis. BMJ Clin Evid. 2015;2015:1111. PMC2907928 19450330

[2] Taunton JE, Ryan MB, Clement DB, McKenzie DC, Lloyd-Smith DR. Plantar fasciitis: a retrospective analysis of 267 cases. Phys Ther Sport. 2002;3(2):57–65. 10.1054/ptsp.2001.0082

[3] Wolgin M, Cook C, Graham C, Mauldin D. Conservative treatment of plantar heel pain - long-term follow-up. Foot Ankle Int. 1994;15(3):97–102. 10.1177/107110079401500303 7951946

[4] Landorf KB, Keenan AM, Herbert RD. Effectiveness of foot orthoses to treat plantar fasciitis: a randomized trial. Arch Intern Med. 2006;166(12):1305–1310. 10.1001/archinte.166.12.1305 16801514

[5] Wang C, Wang F, Yang KD, Weng L, Ko J. Long-term results of extracorporeal shockwave treatment for plantar fasciitis. Am J Sports Med. 2006;34(4):592–596. 10.1177/0363546505281811 16556754

[6] Ibrahim MI, Donatelli RA, Hellman M, Hussein AZ, Furia JP, Schmitz C. Long-term results of radial extracorporeal shock wave treatment for chronic plantar fasciopathy: a prospective, randomized, placebo-controlled trial with two years follow-up. J Orthop Res. 2017;35(7):1532–1538. 10.1002/jor.23403 27567022

[7] Hansen L, Krogh TP, Ellingsen T, Bolvig L, Fredberg U. Long-term prognosis of plantar fasciitis a 5- to 15-year follow-up study of 174 patients with ultrasound examination. Orthop J Sports Med. 2018;6(3):2325967118757983. 10.1177/2325967118757983 PMC5844527 29536022

[8] Rogers J, Jones G, Cook JL, Wills K, Lahham A, Winzenberg TM. Chronic plantar heel pain is principally associated with waist girth (systemic) and pain (central) factors, not foot factors: a case-control study. J Orthop Sports Phys Ther. 2021;51(9):449–458. 10.2519/jospt.2021.10018 33962520

[9] Kosek E, Cohen M, Baron R, et al. Do we need a third mechanistic descriptor for chronic pain states? Pain. 2016;157(7):1382–1386. 10.1097/j.pain.0000000000000507 26835783

[10] Baxter DE, Pfeffer GB, Thigpen M. Chronic heel pain. Treatment rationale. Orthop Clin North Am. 1989;20(4):563–569. 2797751

[11] Freynhagen R, Baron R, Gockel U, Tolle TR. painDETECT: a new screening questionnaire to identify neuropathic components in patients with back pain. Curr Med Res Opin. 2006;22(10):1911–1920. 10.1185/030079906X132488 17022849

[12] Bennett PJ, Patterson C, Wearing S, Baglioni T. Development and validation of a questionnaire designed to measure foot-health status. J Am Podiatr Med Assoc. 1998;88(9):419–428. 10.7547/87507315-88-9-419 9770933

[13] Richardson JDN, Peacock S, Iezzi A. Measurement of the quality of life for economic evaluation and the assessment of quality of life (AQoL) mark 2 instrument. Aust Econ Rev. 2004;37(1):62–88. 10.1111/j.1467-8462.2004.00308.x

[14] Marfell-Jones M, Olds T, Stewart A, Carter L. *International standards for anthropometric assessment* International Society for the Advancement of Kinanthropometry (ISAK), 2006; Potchefstroom, South Africa.

[15] Moraux A, Canal A, Ollivier G, et al. Ankle dorsi- and plantar-flexion torques measured by dynamometry in healthy subjects from 5 to 80 years. BMC Musculoskelet Disord. 2013;14:104. 10.1186/1471-2474-14-104 23522186 PMC3617997

[16] Sullivan MJL, Bishop S, Pivik J. The pain catastrophizing scale: development and validation. Psychol Assess. 1995;7(4):524–532. 10.1037/1040-3590.7.4.524

[17] Sullivan MJ, Adams H, Rhodenizer T, Stanish WD. A psychosocial risk factor--targeted intervention for the prevention of chronic pain and disability following whiplash injury. Phys Ther. 2006;86(1):8–18. 10.1093/ptj/86.1.8 16386058

[18] Bennell KL, Talbot RC, Wajswelner H, Techovanich W, Kelly DH, Hall AJ. Intra-rater and inter-rater reliability of a weight-bearing lunge measure of ankle dorsiflexion. Aust J Physiother. 1998; 44(3):175–180. 10.1016/S0004-9514(14)60377-9 11676731

[19] Hopson MM, Mcpoil TG, Cornwall MW. Motion of the first metatarsophalangeal joint - reliability and validity of 4 measurement techniques. J Am Podiatr Med Assoc. 1995;85(4):198–204. 10.7547/87507315-85-4-198 7738816

[20] Spitzer RL, Kroenke K, Williams JB. Validation and utility of a self-report version of PRIME-MD: the PHQ primary care study. Primary care evaluation of mental disorders. Patient health questionnaire. JAMA. 1999;282(18):1737–1744. 10.1001/jama.282.18.1737 10568646

[21] Singer JD, and Willett JB. Applied Longitudinal Data Analysis: Modeling Change and Event Occurrence. Oxford University Press, 2003; New York. 10.1093/acprof:oso/9780195152968.001.0001

[22] Morrissey D, Cotchett M, Said J'Bari A, et al. Management of plantar heel pain: a best practice guide informed by a systematic review, expert clinical reasoning and patient values. Br J Sports Med. 2021;55(19):1106–1118. 10.1136/bjsports-2019-101970 33785535 PMC8458083

[23] Landorf KB, Radford JA, Hudson S. Minimal important difference (MID) of two commonly used outcome measures for foot problems. J Foot Ankle Res. 2010;3(1):7. 10.1186/1757-1146-3-7 20465855 PMC2881906

[24] Kroenke K, Spitzer RL, Williams JB. The PHQ-9: validity of a brief depression severity measure. J Gen Intern Med. 2001;16(9):606–613. 10.1046/j.1525-1497.2001.016009606.x 11556941 PMC1495268

[25] Slepian P, Bernier E, Scott W, Niederstrasser NG, Wideman T, Sullivan M. Changes in pain catastrophizing following physical therapy for musculoskeletal injury: the influence of depressive and post-traumatic stress symptoms. J Occup Rehabil. 2014;24(1):22–31. 10.1007/s10926-013-9432-2 23529509

[26] Alshami AM, Souvlis T, Coppieters MW. A review of plantar heel pain of neural origin: differential diagnosis and management. Man Ther. 2008;13(2):103–111. 10.1016/j.math.2007.01.014 17400020

[27] Kosek E, Clauw D, Nijs J, et al. Chronic nociplastic pain affecting the musculoskeletal system: clinical criteria and grading system. Pain. 2021;162(11):2629–2634. 10.1097/j.pain.0000000000002324 33974577

[28] Neblett R, Cohen H, Choi Y, et al. The central sensitization inventory (CSI): establishing clinically significant values for identifying central sensitivity syndromes in an outpatient chronic pain sample. J Pain. 2013;14(5):438–445. 10.1016/j.jpain.2012.11.012 23490634 PMC3644381

[29] Wheeler PC . Up to a quarter of patients with certain chronic recalcitrant tendinopathies may have central sensitisation: a prospective cohort of more than 300 patients. Br J Pain. 2019;13(3):137–144. 10.1177/2049463718800352 31308939 PMC6613072

[30] Wheeler PC . Neuropathic pain may be common in chronic lower limb tendinopathy: a prospective cohort study. Br J Pain. 2017;11(1):16–22. 10.1177/2049463716680560 28386400 PMC5370628

[31] Drake C, Mallows A, Littlewood C. Psychosocial variables and presence, severity and prognosis of plantar heel pain: a systematic review of cross-sectional and prognostic associations. Musculoskeletal Care. 2018;16(3):329–338. 10.1002/msc.1246 29766646

[32] Martinez-Calderon J, Jensen MP, Morales-Asencio JM, Luque-Suarez A. Pain catastrophizing and function In individuals with chronic musculoskeletal pain: a systematic review and meta-analysis. Clin J Pain. 2019;35(3):279–293. 10.1097/AJP.0000000000000676 30664551

[33] Picavet HS, Vlaeyen JW, Schouten JS. Pain catastrophizing and kinesiophobia: predictors of chronic low back pain. Am J Epidemiol. 2002;156(11):1028–1034. 10.1093/aje/kwf136 12446259

[34] Lee H, McAuley JH, Hubscher M, Kamper SJ, Traeger AC, Moseley GL. Does changing pain-related knowledge reduce pain and improve function through changes in catastrophizing? Pain. 2016;157(4):922–930. 10.1097/j.pain.0000000000000472 26761387

[35] Liu G, Liang KY. Sample size calculations for studies with correlated observations. Biometrics. 1997;53(3):937–947. 10.2307/2533554 9290224

[36] Muller KE, Lavange LM, Ramey SL, Ramey CT. Power calculations for general linear multivariate models including repeated measures applications. J Am Stat Assoc. 1992;87(420):1209–1226. 10.1080/01621459.1992.10476281 24790282 PMC4002049

[37] Faul F, Erdfelder E, Lang AG, Buchner A. G*power 3: a flexible statistical power analysis program for the social, behavioral, and biomedical sciences. Behav Res Methods. 2007;39(2):175–191. 10.3758/bf03193146 17695343

[38] Evers AWM, Colloca L, Blease C, et al. Implications of placebo and nocebo effects for clinical practice: expert consensus. Psychother Psychosom. 2018;87(4):204–210. 10.1159/000490354 29895014 PMC6191882

