# Supplementary material for: Pain Catastrophizing Beliefs and Neuropathic Symptoms Are Associated With a Poorer Long-Term Recovery in Chronic Plantar Heel Pain: A Cohort Study
Source: Phys Ther. 2025 Nov 7;105(12):pzaf134. doi: 10.1093/ptj/pzaf134 (PMC12708338; doi:10.1093/ptj/pzaf134)
Supplement: PTJ-2025-0165_R2-Supplementary-Material_JR_pzaf134 [file ptj-2025-0165_r2-supplementary-material_jr_pzaf134.pdf]

Supplementary Table 1. Characteristics of missing data.

	Baseline Mean (SD)	Follow-up Mean (SD)	Lost to follow-up Mean (SD)	N missing
Female sex % (n)	60 (131)	60 (126)	50 (5)	10
Age (years)	54.78 (12.13)	55.98 (12.04)	52.71 (13.33)	10
BMI (kg/ m^2)	29.1 (5.4)	29.5 (5.5)	29.48 (5.97)	18
FHSQ pain (/100)	48.8 (21.6)	75.9 (23.3)	51.81 (23.18)	10
FHSQ function (/100)	65.7 (27.8)	86.13 (22.3)	55.63 (34.54)	10
AQoL (/100)	76.4 (10.8)	81.6 (9.7)	68 (11.98)	11
Pain catastrophizing scale (/52)	11.6 (10.9)	6.4 (8.8)	22 (10.54)	11
painDETECT (/38)	9.64 (5.93)	5.04 (6.17)	13.8 (7.76)	10
PHQ-9 (/27)	3.67 (4.01)	2.97 (3.29)	4.40 (2.32)	11

SD standard deviation, BMI body mass index, FHSQ Foot health status questionnaire, AQoL Assessment of quality of life-6D, PHQ-9 Patient health questionnaire

Supplementary Table 2. Other factors: full multivariable models for the outcomes of pain, function and quality of life.

**Full multivariable model**[a]		**FHSQ pain**[b]			**FHSQ function**[c]			**AQOL**[d]		
		β	95% CI		β	95% CI		β	95% CI	
Ankle DF ROM, knee flexed (degrees)	main effect[e]	**0.44**	0.01	0.88	**0.29**	-0.22	0.81	**0.18**	0.00	0.35
	time[e]	**51.15**	24.14	78.16	**40.90**	17.35	64.44	**11.71**	4.27	19.16
	time interaction[f]	*-0.57*	-1.18	0.04	*-0.51*	-1.05	0.04	*-0.15*	-0.33	0.02
1st MTPJ Extension ROM (degrees)	main effect	0.05	-0.15	0.24	0.04	-0.20	0.29	0.03	-0.05	0.11
	time	**23.56**	6.93	40.19	**20.30**	3.80	36.81	3.03	-1.31	7.37
	time interaction	0.04	-0.19	0.27	-0.02	-0.24	0.20	0.03	-0.03	0.09
Straight leg raise ROM (degrees)	main effect	0.03	-0.20	0.25	**0.31**	0.08	0.54	**0.12**	0.02	0.21
	time	**25.42**	7.69	43.14	**30.33**	14.30	46.37	**6.02**	1.38	10.65
	time interaction	0.01	-0.22	0.25	-0.16	-0.37	0.05	-0.02	-0.08	0.04
Moderate to vigorous physical activity (average mins/day)	main effect	0.07	-0.02	0.15	**0.15**	0.03	0.26	**0.05**	0.02	0.08
	time	**30.16**	24.31	36.01	**24.00**	17.85	30.16	**6.70**	5.01	8.38
	time interaction	-0.09	-0.20	0.02	*-0.12*	-0.25	0.01	**-0.04**	-0.07	-0.01
Average steps/day (/1000 steps)	main effect	0.52	-0.39	1.42	**1.94**	0.58	3.30	**0.63**	0.28	0.99
	time	**34.52**	24.42	44.61	**31.31**	19.45	43.17	**5.67**	2.53	8.82
	time interaction	-0.96	-2.16	0.25	**-1.50**	-2.89	-0.11	-0.09	-0.43	0.24
Average sedentary minutes/ day	main effect	0.03	-0.01	0.06	**0.05**	0.01	0.09	0.00	-0.01	0.02
	time	**26.78**	4.19	49.37	**31.12**	7.09	55.16	**6.57**	0.43	12.71
	time interaction	0.00	-0.05	0.04	-0.02	-0.07	0.02	0.00	-0.02	0.01
Morning stiffness (Y/N, ref. no morning stiffness)[g]	main effect	-4.60	-11.69	2.48	**-12.00**	-19.53	-4.47	**-3.99**	-6.36	-1.62
	time	**26.86**	22.81	30.92	**17.75**	13.83	21.68	**4.87**	3.78	5.95
	time interaction	-9.00	-20.40	2.41	1.16	-10.31	12.63	-2.13	-5.39	1.13
Depression (PHQ9)[h]	main effect	**-1.29**	-2.21	-0.37	**-3.25**	-4.04	-2.46	**-1.46**	-1.74	-1.18
	time	**28.75**	23.56	33.94	**15.28**	10.95	19.62	**3.90**	2.60	5.21
	time interaction	-1.15	-2.65	0.35	0.59	-0.53	1.71	0.09	-0.35	0.54

CI confidence interval, DF dorsiflexion, MTPJ metatarsophalangeal joint, ROM range of motion, PHQ9 Patient Health Questionnaire-9 item

[a] inverse probability weighted for missing data, bold significant p<0.05, italic p<0.10. All models adjusted for age, sex, time, comorbidities and current smoking status, and non-physical activity factors also adjusted for physical activity (average moderate to vigorous PA minute/day).

[b] Foot health status questionnaire pain, 0-100, higher is less pain
[c] Foot health status questionnaire function, 0-100, higher is better function
[d] Assessment of Quality of Life, 0-100, higher is better quality of life
[e] conditional main effect for exposure and time, respectively
[f] interaction term of exposure with time
[g] do you have morning stiffness at any joint/s last longer than 30 minutes? (yes/ no)
[h] Patient Health Questionnaire 9, 0-52, higher is more depressed

Supplementary Figures 1–3. Exposure-time interaction plots for full multivariable model.

Pain

1a.

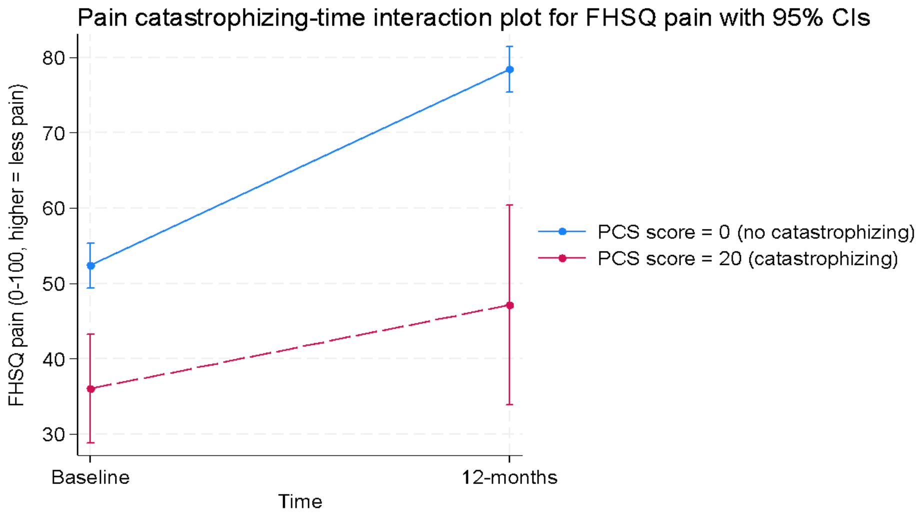

1b.

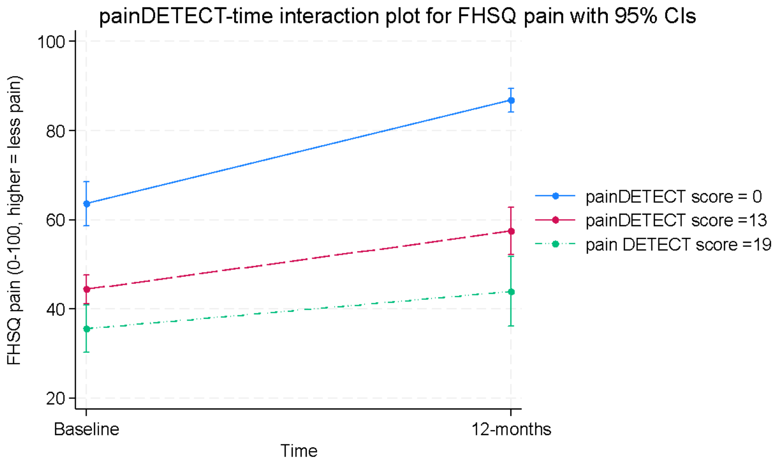

1c.

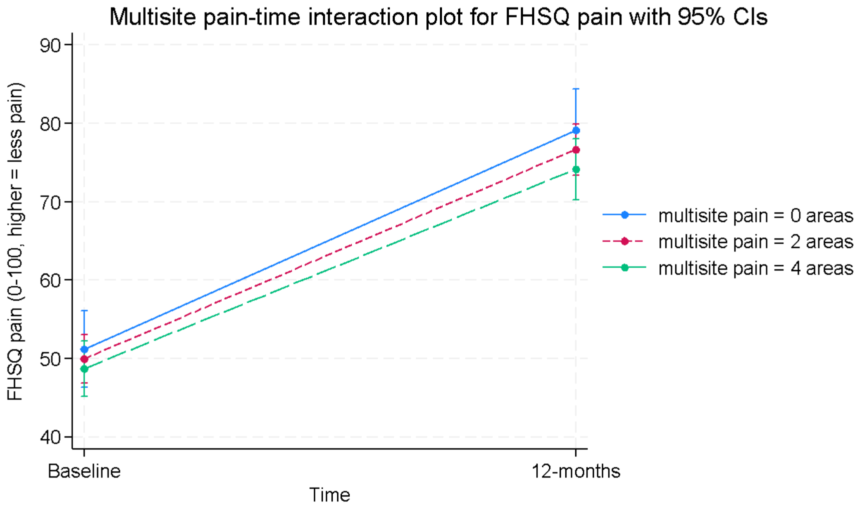

Multisite pain-time interaction plot for FHSQ pain with 95% CIs
FHSQ pain (0-100, higher = less pain)
90
80
70
60
50
40
Baseline
12-months
Time
multisite pain = 0 areas
multisite pain = 2 areas
multisite pain = 4 areas

1d.

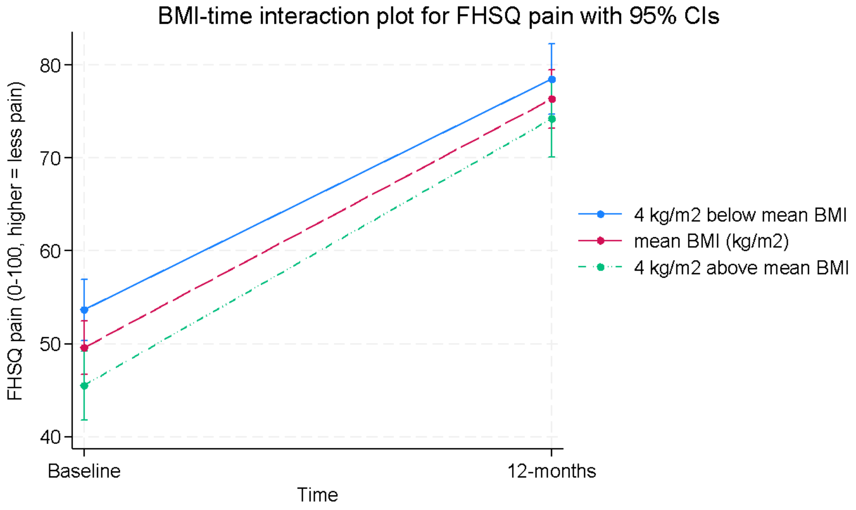

BMI-time interaction plot for FHSQ pain with 95% CIs
FHSQ pain (0-100, higher = less pain)
80
70
60
50
40
Baseline
12-months
Time
4 kg/m2 below mean BMI
mean BMI (kg/m2)
4 kg/m2 above mean BMI

1e.

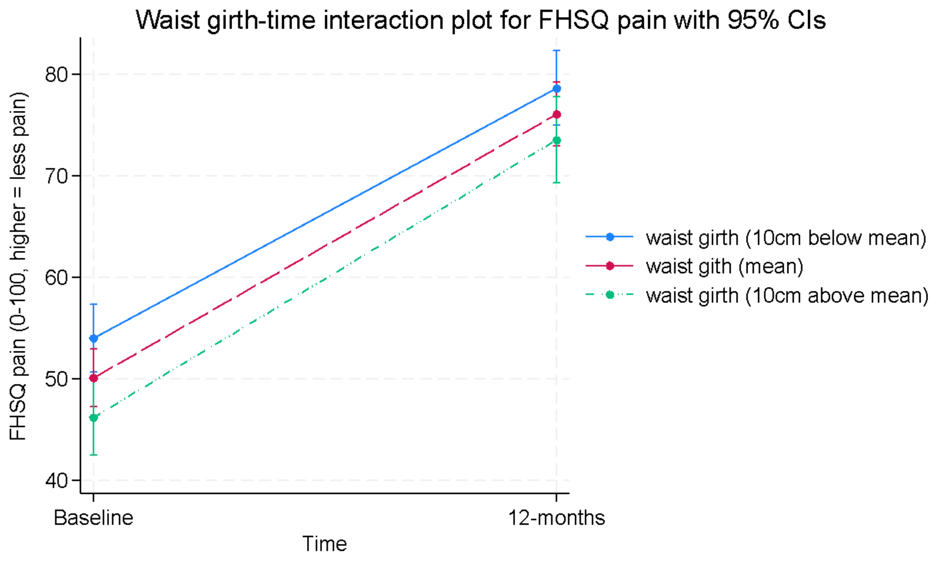

1f.

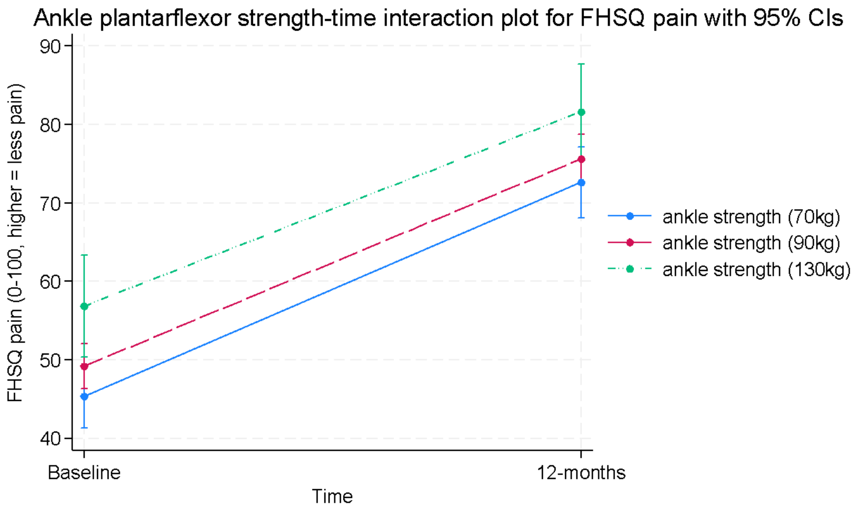

Figure 1.

Exposure-time interaction plots for outcome of pain; full multivariable model adjusted for age, sex, time, co-morbidities, current smoking status and physical activity. All exposures of interest modelled in continuous form, plotted at nominated levels.

1(a) Pain catastrophizing beliefs. Trajectory of pain outcomes for sub-groups with no pain catastrophizing beliefs (pain catastrophizing score=0), versus those classified as a clinical catastrophizing (PCS=20).

1(b) painDETECT (neuropathic pain score). Trajectory of pain outcomes for sub-groups whose painDETECT score indicates either no neuropathic symptoms (0), 'probably neuropathic' (19), or somewhere in between (score =13).

1(c) Multisite pain. Trajectory of pain outcomes for sub-groups with either no other site of pain beyond the heel, or 2 or 4 other sites.

1(d) BMI. Trajectory of pain outcomes for sub-groups with a BMI +/- 4 kg/m^2 from the cohort mean (approximately 25th, 50th and 75th percentiles).

1(e) Waist girth. Trajectory of pain outcomes for sub-groups with a waist girth +/- 10cm from the cohort mean (approximately 25th, 50th and 75th percentiles).

1(f) Ankle plantar flexor strength. Trajectory of pain outcomes for subgroups with ankle strength at 70, 90 and 130kg. This model also adjusted for body weight.

Function

2a.

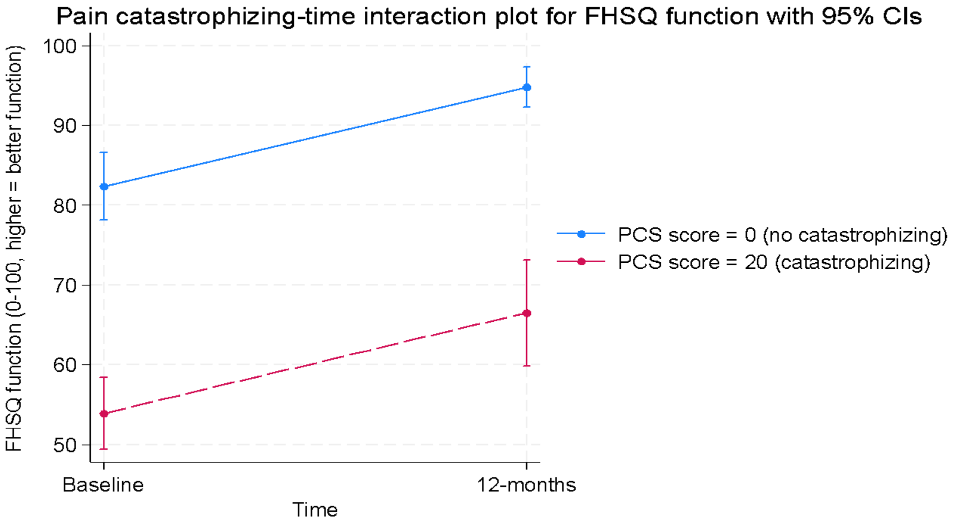

2b.

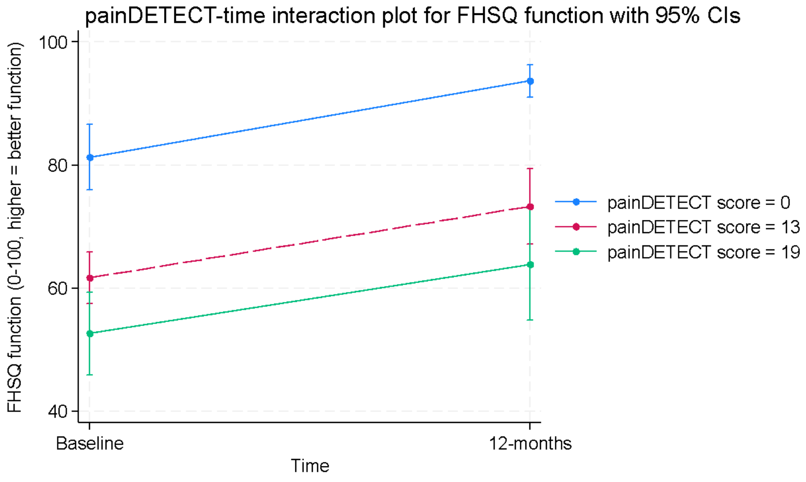

.

2c.

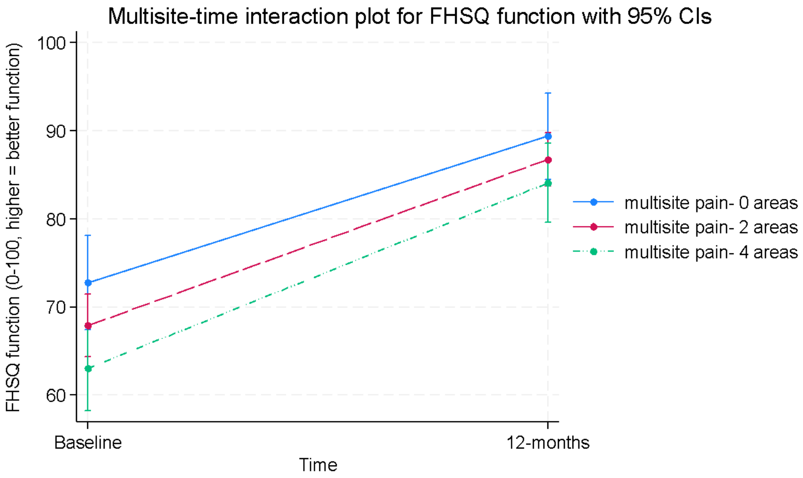

Multisite-time interaction plot for FHSQ function with 95% CIs
FHSQ function (0-100, higher = better function)
100
90
80
70
60
Baseline
12-months
Time
multisite pain- 0 areas
multisite pain- 2 areas
multisite pain- 4 areas

2d.

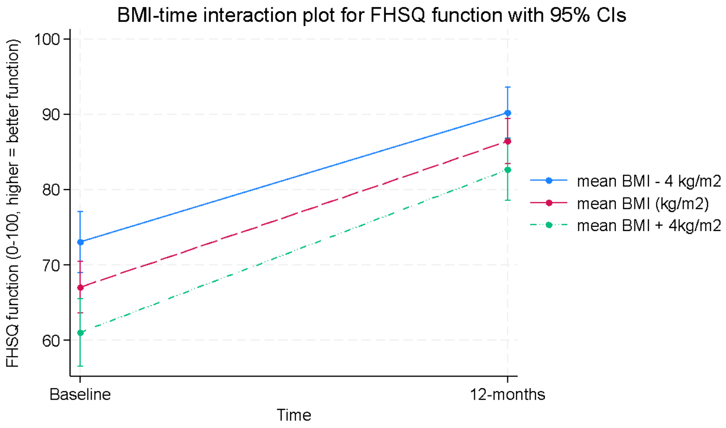

BMI-time interaction plot for FHSQ function with 95% CIs
FHSQ function (0-100, higher = better function)
100
90
80
70
60
Baseline
12-months
Time
mean BMI - 4 kg/m2
mean BMI (kg/m2)
mean BMI + 4kg/m2

2e.

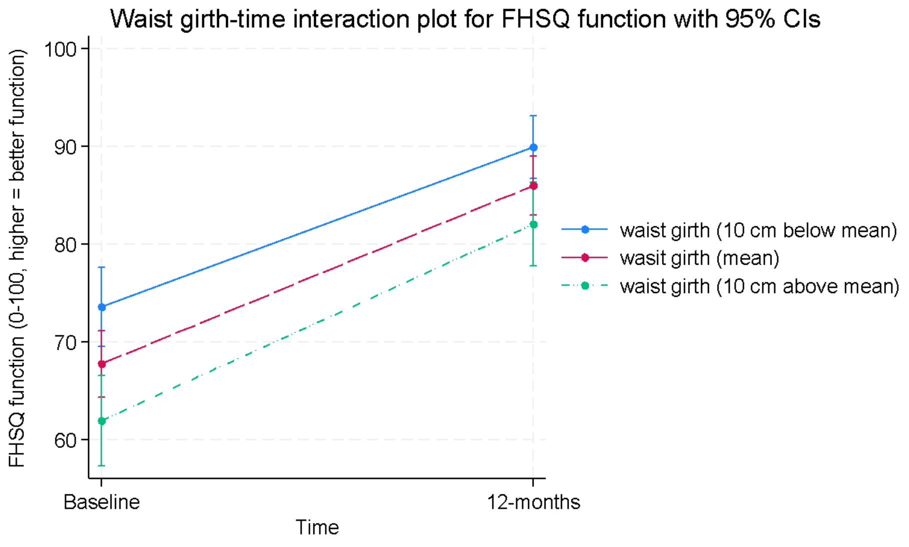

2f.

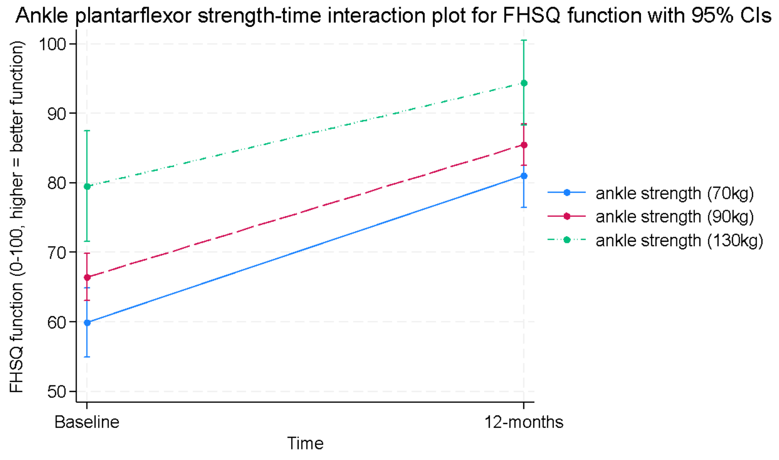

Figure 2.

Exposure-time interaction plots for outcome of function; full multivariable model adjusted for age, sex, time, co-morbidities, current smoking status and physical activity. All exposures of interest modelled in continuous form, at nominated levels.

2(a) Pain catastrophizing beliefs. Trajectory of pain outcomes for sub-groups with no pain catastrophizing beliefs (pain catastrophizing score=0), versus those classified as clinically catastrophizing (PCS=20).

2(b) painDETECT (neuropathic pain score). Trajectory of pain outcomes for sub-groups whose painDETECT score indicates either no neuropathic symptoms (0), ‘probably neuropathic’ (19), or somewhere in between (score =13).

2(c) Multisite pain. Trajectory of pain outcomes for sub-groups with either no other site of pain beyond the heel, or 2 or 4 other sites.

2(d) BMI. Trajectory of pain outcomes for sub-groups with a BMI +/- 4 kg/m^2 from the cohort mean (approximately 25th, 50th and 75th percentiles).

2(e) Waist girth. Trajectory of pain outcomes for sub-groups with a waist girth +/- 10cm from the cohort mean (approximately 25th, 50th and 75th percentiles).

2(f) Ankle plantar flexor strength. Trajectory of pain outcomes for subgroups with ankle strength at 70, 90 and 130kg. This model also adjusted for body weight.

Quality of life

3a.

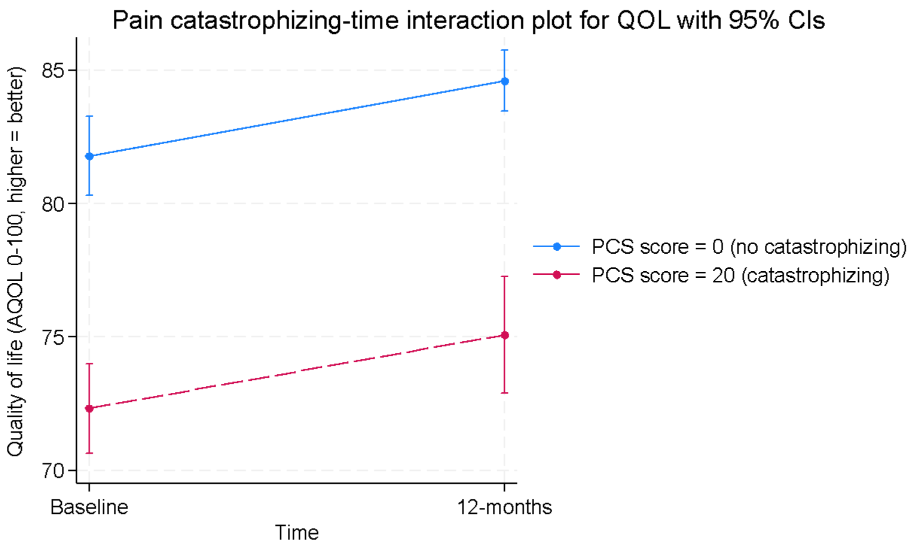

3b.

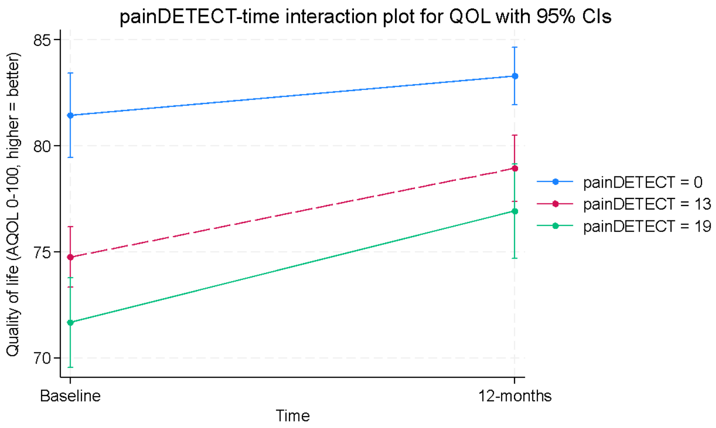

3c.

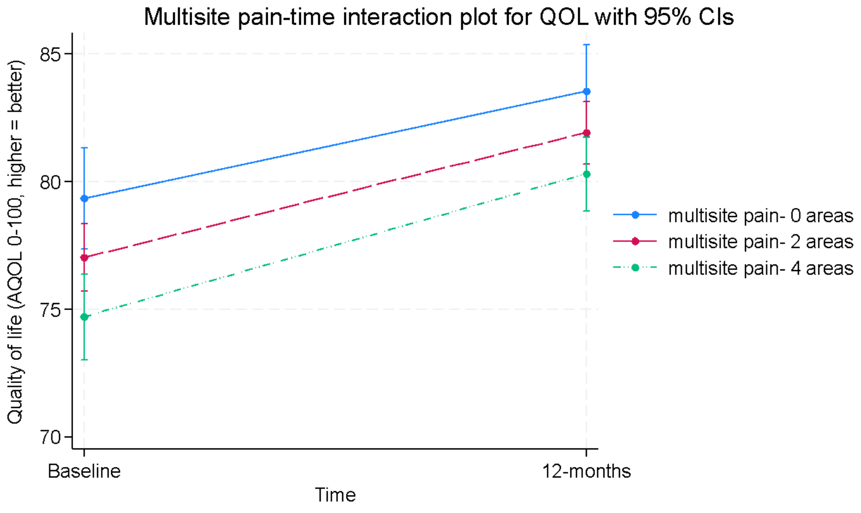
Multisite pain-time interaction plot for QOL with 95% CIs
Quality of life (AQOL 0-100, higher = better)
85
80
75
70
Baseline
12-months
Time
multisite pain- 0 areas
multisite pain- 2 areas
multisite pain- 4 areas

3d.

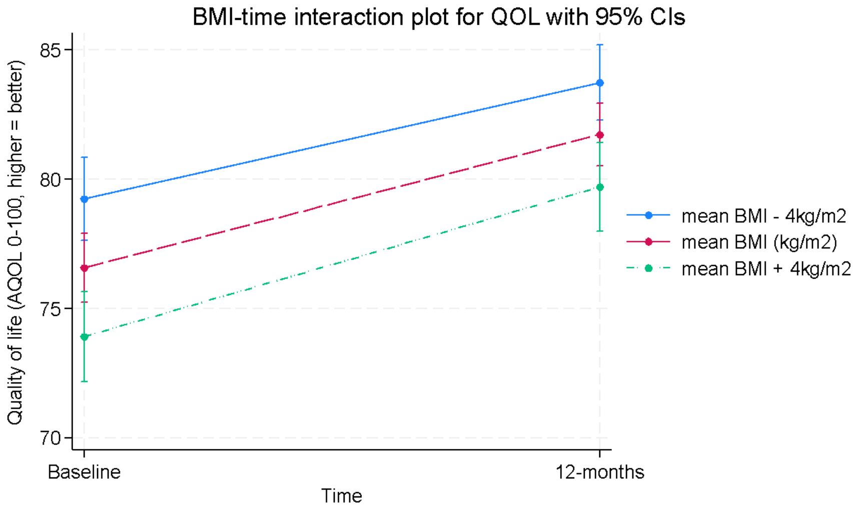
BMI-time interaction plot for QOL with 95% CIs
Quality of life (AQOL 0-100, higher = better)
85
80
75
70
Baseline
12-months
Time
mean BMI - 4kg/m2
mean BMI (kg/m2)
mean BMI + 4kg/m2

3e.

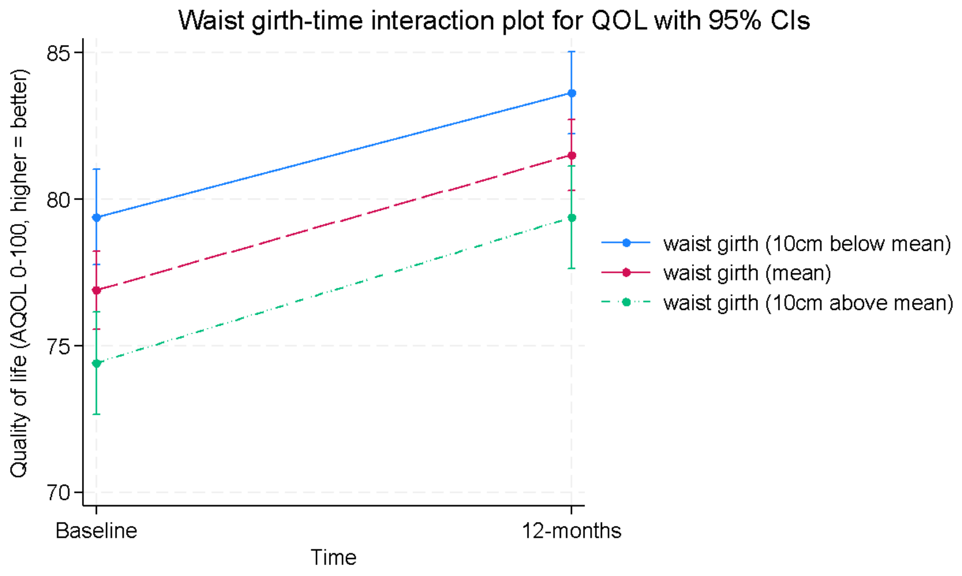

3f.

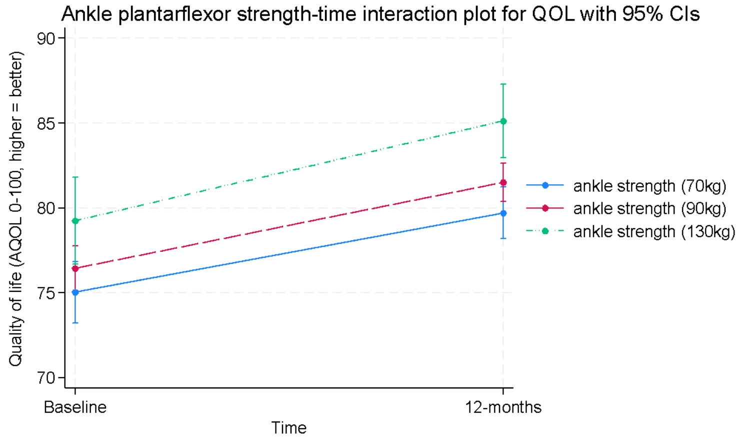

Figure 3.

Exposure-time interaction plots for outcome of quality of life; full multivariable model adjusted for age, sex, time, co-morbidities, current smoking status and physical activity. All exposures of interest modelled in continuous form, plotted at nominated levels.

3(a) Pain catastrophizing beliefs. Trajectory of pain outcomes for sub-groups with no pain catastrophizing beliefs (pain catastrophizing score=0), versus those classified as a clinical catastrophizer (PCS=20).

3(b) painDETECT (neuropathic pain score). Trajectory of pain outcomes for sub-groups whose painDETECT score indicates either no neuropathic symptoms (0), 'probably neuropathic' (19), or somewhere in between (score =13).

3(c) Multisite pain. Trajectory of pain outcomes for sub-groups with either no other site of pain beyond the heel, or 2 or 4 other sites.

3(d) BMI. Trajectory of pain outcomes for sub-groups with a BMI +/- 4 kg/m^2 from the cohort mean (approximately 25th, 50th and 75th percentiles).

3(e) Waist girth. Trajectory of pain outcomes for sub-groups with a waist girth +/- 10cm from the cohort mean (approximately 25th, 50th and 75th percentiles).

3(f) Ankle plantar flexor strength. Trajectory of pain outcomes for subgroups with ankle strength at 70, 90 and 130kg. This model also adjusted for body weight.

Supplementary Figure 4

a.

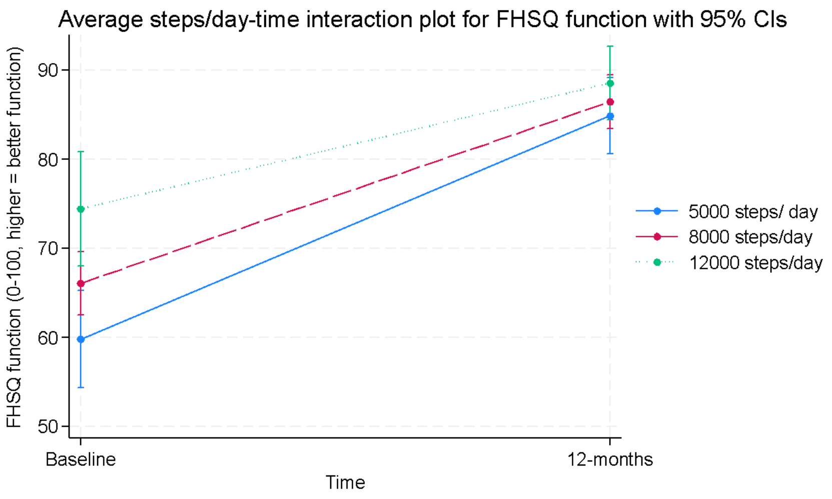

b.

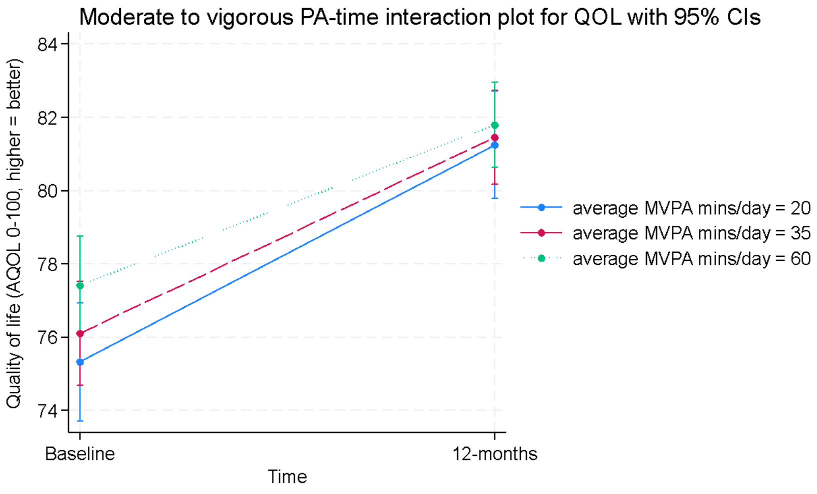

Figure 4. Exposure-time interaction plots for outcomes of function and quality of life; full multivariable model adjusted for age, sex, time, co-morbidities, and current smoking status. Exposures of interest modelled in continuous form, plotted at nominated levels.

(a) Average steps/day for outcome of function. Trajectory of outcome for subgroups who average 5000, 8000 or 12000 steps per day.

(b) Average moderate to vigorous minutes/day for outcome of quality of life. Trajectory of outcome for sub-groups who average 20, 35 or 60 MVPA minutes/ day. These points reflect the 25th, 50th and 75th percentile for this variable. (MVPA moderate to vigorous physical activity) score increased (worse depressive symptoms) or decreased by one standard deviation (4 points) or stayed the same.
